# Isolation and Identification of a New Isolate of Anguillid Herpesvirus 1 from Farmed American Eels (*Anguilla rostrata*) in China

**DOI:** 10.3390/v14122722

**Published:** 2022-12-07

**Authors:** Rui Guo, Zheng Zhang, Tianliang He, Miaomiao Li, Yuchen Zhuo, Xiaoqiang Yang, Haiping Fan, Xinhua Chen

**Affiliations:** 1 Key Laboratory of Marine Biotechnology of Fujian Province, College of Marine Sciences, College of Life Sciences, Fujian Agriculture and Forestry University, Fuzhou 350002, China; 2 Fuzhou Ocean and Fisheries Technology Center, Fuzhou 350007, China; 3 Fujian Provincial Fishery Technical Extension Center, Fuzhou 350002, China; 4 Freshwater Fisheries Research Institute of Fujian Province, Fuzhou 350002, China; 5 Southern Marine Science and Engineering Guangdong Laboratory (Zhuhai), Zhuhai 519000, China

**Keywords:** Anguillid herpesvirus 1, AngHV-1-FC, American eel (*Anguilla rostrate*), pathogenicity, genomic analysis

## Abstract

Anguillid herpesvirus 1 (AngHV-1) is a pathogen that causes hemorrhagic disease in various farmed and wild freshwater eel species, resulting in significant economic losses. Although AngHV-1 has been detected in the American eel (*Anguilla rostrata*), its pathogenicity has not been well characterized. In this study, an AngHV-1 isolate, tentatively named AngHV-1-FC, was isolated from diseased American eels with similar symptoms as those observed in AngHV-1-infected European eels and Japanese eels. AngHV-1-FC induced severe cytopathic effects in the European eel spleen cell line (EES), and numerous concentric circular virions were observed in the infected EES cells by transmission electron microscopy. Moreover, AngHV-1-FC caused the same symptoms as the naturally diseased European eels and Japanese eels through experimental infection, resulting in a 100% morbidity rate and 13.3% mortality rate. The whole genome sequence analyses showed that the average nucleotide identity value between AngHV-1-FC and other AngHV-1 isolates ranged from 99.28% to 99.55%. However, phylogenetic analysis revealed that there was a genetic divergence between AngHV-1-FC and other AngHV-1 isolates, suggesting that AngHV-1-FC was a new isolate of AngHV-1. Thus, our results indicated that AngHV-1-FC can infect farmed American eels, with a high pathogenicity, providing new knowledge in regard to the prevalence and prevention of AngHV-1.

## 1. Introduction

Eel is one of the most important freshwater farmed fish in China. At present, the farmed species of eels include the European eel (*Anguilla anguilla*), Japanese eel (*A. japonica*), and American eel (*A. rostrata*). Given that the seeding production of European eels and Japanese eels has decreased in recent years, American eels have become the largest farmed eel species in China [1]. Since the 1980s, the occurrence of hemorrhagic infectious disease has become common in farmed European eels and Japanese eels, causing hemorrhaging in the skin, fins, gills, and liver, and resulting in mass mortality [2]. This disease has been observed in almost all wild and farmed species of eels [3,4]. In China, the annual outbreak of hemorrhagic disease in farmed eels has resulted in a high mortality rate of up to 30%, leading to huge economic losses [5].

Anguillid herpesvirus 1 (AngHV-1) has been identified as the pathogen that causes hemorrhagic disease, and it was first isolated from diseased European eels and Japanese eels in 1985 [6,7]. It has also been detected in American eels, shortfin eels (*A. bicolor bicolor*) and giant mottled eels (*A. marmorata*) [8,9,10]. AngHV-1 can infect naive eels by intraperitoneal injection, which indicates that it is highly virulent [11]. However, it has also been reported that AngHV-1 can establish itself as a latent infection in European eels without clinical signs and death, indicating that there are differences in the pathogenicity of different AngHV-1 isolates [12,13]. AngHV-1 is a member of the genus *Cyprinivirus* in the *Alloherpesviridae* family, and contains an approximately 248.5 kb linear double-stranded DNA (dsDNA) genome encoding 129 open reading frames (ORFs) [14,15,16]. A recent study sequenced seven AngHV-1 isolates from European eels of various geographical origin and compared their genetic properties with two other reported genomes of the Japanese eel and European eel. The results showed the differences in the genome sequence and proliferative capacity in vitro between the nine AngHV-1 isolates [17]. However, it is unclear whether these differences exist in the AngHV-1 isolate from the American eel.

In this study, we isolated and identified an isolate of AngHV-1, tentatively named AngHV-1-FC, from farmed American eels with hemorrhagic disease. The pathogenicity of AngHV-1-FC in the American eel was investigated by experimental infection. In addition, we sequenced the genome of AngHV-1-FC and performed a comparative genome analysis of AngHV-1-FC and other reported AngHV-1 isolates. Our data indicated that AngHV-1-FC is a new isolate of AngHV-1 from the farmed American eel in China, thus providing new knowledge in regard to the prevalence and prevention of AngHV-1.

## 2. Materials and Methods

### 2.1. Sample Collection

The farmed American eels were visually inspected for clinical symptoms in a warm-water pond in Longyan, China. A total of ten moribund fish (averaging ~25 g in weight) were anesthetized with Tricaine-S (Sigma-Aldrich, St. Louis, MO, USA) and randomly anatomized to examine the clinical symptoms. The gills, liver, and spleen tissues were collected for the following analysis.

### 2.2. Virus Detection

A total of 20 mg of the tissue collected from the fish was homogenized to extract viral DNA using an EasyPure Viral DNA/RNA Kit (TransGen Biotech, Beijing, China) following the manufacturer’s instructions. The DNA polymerase gene of AngHV-1 (Accession no. AF333066.1) was detected by PCR using the primer sets (forward primer: 5′-GTGTCGGGCCTTTGTGGTGA-3′ and reverse primer: 5′-CATGCCGGGAGTCTTTTTGAT-3′) [18]. PCR amplifications were performed using the Premix Taq (Takara, Dalian, China) under the following conditions: pre-denaturation at 94 °C for 5 min, 32 cycles of denaturation at 94 °C for 30 s, annealing at 60 °C for 25 s and extension at 72 °C for 30 s, with a final extension at 72 °C for 5 min. The PCR products were examined on agarose gel electrophoresis and purified for sequencing at Sangon Biotech (Shanghai) Co., Ltd.

### 2.3. Cell Culture

The EES cell line was established according to previously described methods [19,20]. Briefly, the spleen of the European eel was digested with trypsin solution, and the cell suspension was transferred into cell culture flasks and cultured at 28 °C. Cells were serially passaged every 3 days, and grown in DMEM/F12 medium (Hyclone, Logan, UT, USA) supplemented with 100 IU penicillin, 100 μg/mL of streptomycin and 15% of FBS (Gibco, Waltham, MA, USA) at 28 °C. The 50th passage of the EES cell line was used in this study.

### 2.4. Virus Isolation and Purification

The tissues collected from the virus-positive diseased fish above were homogenized in 10 volumes of DMEM/F12 medium and centrifuged at 10,000 g for 5 min. The supernatant was filtered through a 0.45 µm membrane, and then it was inoculated into the EES cells in a concentration of 100 μL filtrate per 10^6^ cells. The inoculated cells were cultured in DMEM/F12 medium with 100 IU penicillin, 100 μg/mL of streptomycin and 3% of FBS at 28 °C. The cytopathic effects (CPE) were observed daily for 7 days. When over 80% cells showed CPE, the suspension was transferred to the sub-passage. The culture supernatant was collected and cell debris was removed by differential centrifugation with 3000, 4500, 6000, 8000, 12,000 and 15,000 g at 10 °C for 30 min. After centrifugation, the supernatant containing viral particles was filtered with a disposable needle filter with a pore size of 0.22 µm to obtain a purified virus solution. Virus titer values were determined using a 50% tissue culture infective dose (TCID_50_) calculated by the modified Reed and Muench method [21].

### 2.5. Morphology Observation of Viruses

The purified virus solution was inoculated into EES cells. When the CPE was more than 60%, the cells were collected and centrifuged at 350 g for 5 min. The residual pellet was washed twice with sterile phosphate-buffered saline (PBS) solution and fixed by 2.5% glutaraldehyde in 0.1 M PBS (pH 7.4) at 4 °C for 2 h. After staining with osmium tetroxide, the cells were embedded and sectioned using an ultramicrotome according to the previously described methods [22]. The viral morphology was observed using a JEOL JEM-1230 or Hitachi-HT7800 transmission electron microscope operating at 80.0 kV.

### 2.6. Experimental Infection and Histopathological Observation

The American eels were purchased from an eel farm in Sanming, China, where eels were confirmed to be free of AngHV-1 by detecting viral DNA polymerase gene by PCR. Referring to our previously published methods [22,23], the healthy American eels (n = 30), with an average weight of 35 g, were divided into two groups (15 fish each) and were acclimatized in separate tanks with aerated fresh water at 28 °C for 2 weeks. The purified virus solution was diluted with PBS to 1.26 × 10^6^ TCID_50_/mL, and the infection group was injected intraperitoneally with 0.1 mL of the virus solution. The control group was injected with the same volume of sterile PBS. The fish were kept separately in a 100 L aquarium at 28 °C and examined for clinical signs and mortality daily. The cumulative mortality rate was calculated based on the data until 28 days post-challenge. Subsequently, AngHV-1-FC was detected in both moribund and surviving fish by PCR. To determine the viral copy number in the fish in the control group or infection group, a quantitative PCR based on the standard curve method was further performed. Briefly, the quantitative PCR was established by using the AngHV-1-FC ORF25 as target gene, and the primer sets (forward primer: 5′-GGCCCCGAAAGCTGTTCC-3′ and reverse primer: 5′- CGGTGGTTTGCAGCCGAA-3′) were designed. A series of dilutions of recombinant plasmid pMD18-T-ORF25 with known copy numbers were used to establish a standard curve. The quantitative PCR was performed using the TB Green Premix Ex Taq (Takara, Dalian, China) on a QuantStudio 5 system (Thermo, Waltham, MA, USA) under the following conditions: pre-denaturation at 95 °C for 30 s, 40 cycles of denaturation at 94 °C for 10 s, annealing at 60 °C for 35 s with the fluorescence in the SYBR channel acquired. The standard curve equation was Y = −3.755 × logX + 39.168 (Y: Ct value; X: copy number), and the viral copy number (X) was calculated by the equation.

Tissue from the gills, liver and spleen of the post-challenge moribund fish with obvious symptoms in the infection group and the same tissues from the healthy fish in the control group were fixed for transmission electron microscope observation as described above. The same samples were also fixed in 4% paraformaldehyde solution for histopathologic examination. The fixed tissues were embedded in paraffin wax, and the sections, with a 4 µm thickness, were cut by a sliding microtome and stained with hematoxylin and eosin (H&E) [22,23]. The sections were observed using a Nikon Eclipse Ci-L microscope with a Nikon digital sight DS-FI2 camera.

### 2.7. Viral Genome Sequencing and Bioinformatic Analysis

The virus was concentrated by ultracentrifugation as described above, and the DNA was also extracted with EasyPure Viral DNA/RNA Kit. Approximately 3 μg of purified total genomic DNA was used for sequencing. The sequencing was performed using TruSeq DNA Nano with Novaseq 6000 (Illumina, San Diego, CA, USA) at Hangzhou Mingke Biotechnology Co., Ltd. Briefly, sequences were individually assembled using metaSPAdes 3.12.0 [24], contigs were linked to scaffolds using Bambus 2 [25], and the open reading frames (ORF) were predicted using Prodigal [26]. The conserved motifs and domains of the putative proteins composed of >50 amino acids were analyzed using the Conserved Domains Database (http://www.ncbi.nlm.nih.gov/Structure/cdd/wrpsb.cgi (accessed on 4 October 2022)). The genome sequence was then submitted to GenBank under accession no. OM649903.1, named AngHV-1-FC. The linear genome structure map was drawn using Adobe Illustrator CS6 and the circular schematic diagram drawn using the BLAST Ring Image Generator (BRIG) 0.95 (http://sourceforge.net/projects/brig/ (accessed on 4 October 2022)). In addition, the average nucleotide identity (ANI) calculation based on BLAST + was performed among the genomes of 10 AngHV isolates and 3 related *Cyprinivirus* isolates (CyHV-1, -2 and -3) using JSpeciesWS (version 3.9.1) [27]. Detailed information regarding the sequences is listed in the Supplementary Materials, Table S1.

### 2.8. Phylogenetic Analysis

For the phylogenetic analysis of core genes, the amino acid sequences of 3 herpesvirus core genes including DNA polymerase, DNA helicase, and terminase genes retrieved from each AngHV-1 and *Cyprinivirus* genome were concatenated. The sets of concatenated sequences were multiple sequences aligned by MAFFT (online) [28] and used to construct the phylogenetic tree using the neighbor-joining method with 1000 bootstrap replicates in MEGA (v11.0.10) [29]. The concatenated sequences of 3 core genes from Ranid herpesvirus 1 (RaHV-1) of *Alloherpesviridae* were used as the outgroup.

For the phylogenetic analysis of whole genomes, 10 AngHV-1 genomes were multiple sequences aligned by MAFFT (online) and a phylogenetic tree was constructed by UPGMA (unweighted pair group method with arithmetic means) in MEGA. Estimates of the evolutionary divergence between the AngHV-1 isolates were calculated using MEGA.

Detailed information regarding all the AngHV-1, *Cyprinivirus* and RaHV-1 genomes used in this study is listed in Supplementary Materials, Table S1.

## 3. Results

### 3.1. Clinical Symptoms and Virus Detection

**Figure 1:**
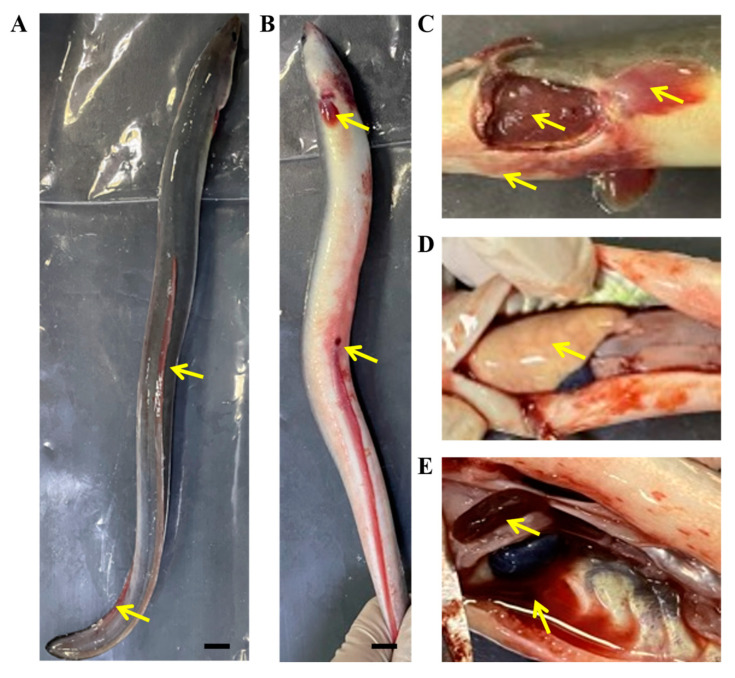
Clinical symptoms of hemorrhagic disease in the naturally diseased American eels. (**A**) Congestion of the dorsal fin. (**B**) Congestion of the pectoral fin and anal fin, and red abdomen and swollen anus. (**C**) Ischemic gill and mandibular hyperemia. (**D**) Ischemic liver. (**E**) Enlarged and congested spleen, abdominal hemorrhage. Yellow arrows show the different tissues with clinical symptoms. Scale bar = 1 cm.

In June 2019, an infectious disease outbreak occurred in farmed American eels (*A. rostrata*) at a water temperature of 28 °C in Longyan, China. The moribund fish had an average weight of 25 g and displayed congestion in the dorsal fin, pectoral fin and anal fin, as well as a red and swollen abdomen and anus (Figure 1A,B). Anatomic analysis showed they had pale gills, bleeding from the abdominal cavity, severe anemia of the liver and enlargement of the spleen (Figure 1C–E). These clinical symptoms were similar to those of hemorrhagic disease caused by AngHV-1 in European eels and Japanese eels [5,6,7,11].

To characterize the pathogen of this disease, a 394 bp segment of DNA polymerase gene of AngHV-1 was amplified from the lesion tissue of moribund fish (Supplementary Materials, Figure S1). After sequencing and alignment analysis, the amplified sequence had a 100% identity with the corresponding sequence of DNA polymerase gene from AngHV-1 (Supplementary Materials, Table S2). These results suggested that AngHV-1 may be the pathogen that caused hemorrhagic disease in the American eels.

### 3.2. Virus Isolation

**Figure 2:**
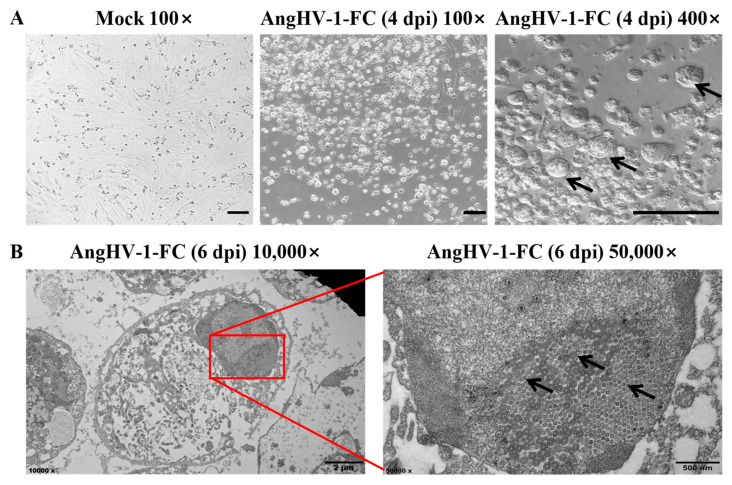
AngHV-1-FC infection in EES cells. (**A**) CPE induced by AngHV-1-FC in EES cells. EES cells were infected with AngHV-1-FC at 4 dpi, and the swollen cells and syncytium were found in 400× magnification. The morphology was observed under light microscope. Black arrows represent the syncytiums, and scale bar = 200 µm. (**B**) The cells with CPE under electron microscope. Numerous concentric circular viral nucleocapsid were observed in the nuclear under electron microscope with 50,000× magnification. Black arrows represent the viral nucleocapsids, red frame represent the detail view, and scale bar = 2 µm or 500 nm.

To isolate the virus that caused hemorrhagic disease in the American eels, the homogenates of lesion tissues were inoculated into the EES cells. After incubation at 28 °C for 4 days, obvious CPE was observed in the infected EES cells. Compared with the control, the numerous infected cells had a round appearance, formed syncytium, and were dislodged from the monolayers (Figure 2A). TEM observation showed a large number of viral nucleocapsids with a morphology of concentric circle (~100 nm in diameter) in the nucleus of EES cells at 4 days post inoculation (dpi) (Figure 2B). After virus isolation and purification, the DNA polymerase gene of the virus isolate was amplified and aligned with the homologous gene of AngHV-1 (Accession no. AF333066.1), showing a 100% identity (Supplementary Materials, Figure S2). Thus, the virus isolate was tentatively named AngHV-1-FC.

### 3.3. Pathogenicity of AngHV-1-FC

**Figure 3:**
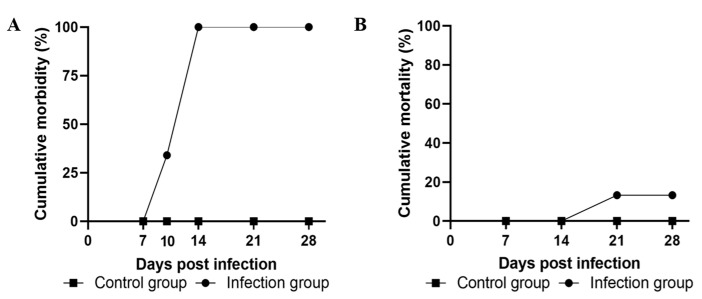
The cumulative morbidity rate and mortality rate of the American eels infected with AngHV-1-FC. (**A**) Cumulative morbidity and (**B**) cumulative mortality of the eels artificially infected AngHV-1-FC. The infection group was injected with 1.26 × 10^5^ TCID_50_ of AngHV-1-FC per fish. The control group was injected with PBS.

The experimental infection was performed to determine the pathogenicity of AngHV-1-FC in American eels. Upon challenge with AngHV-1-FC at a dose of 1.26 × 10^5^ TCID_50_ per fish, similar clinical symptoms including congestion of the anal fin and ischemic liver were observed in the eels in the infection group at 10 dpi (Supplementary Materials, Figure S3A). In contrast, the eels in the control group showed no clinical symptoms of hemorrhagic disease (Supplementary Materials, Figure S3B). The cumulative morbidity of the infection group was 35% at 10 dpi and quickly reached 100% at 14 dpi (Figure 3A). The deaths in the infection group occurred at 21 dpi and the cumulative mortality was 13.3% during 28 dpi, while no deaths were found in the control group (Figure 3B).

**Figure 4:**
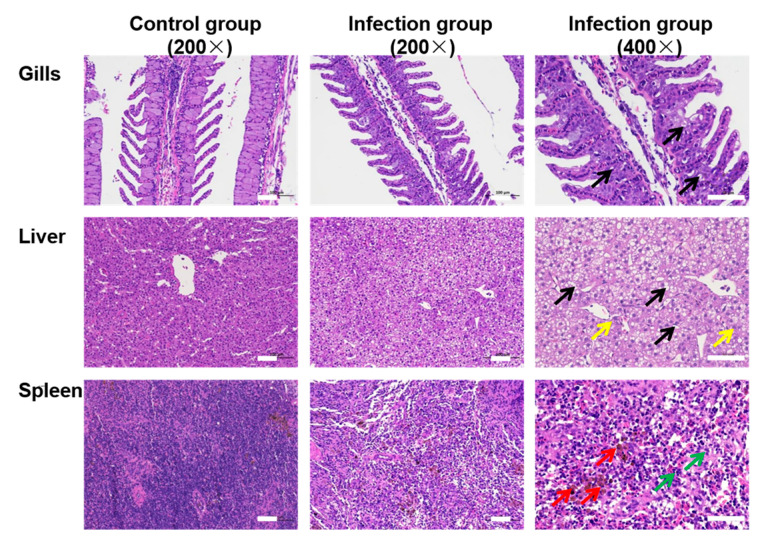
Histopathological examination of tissues from the American eels infected with AngHV-1-FC. All sections were stained with H&E. Black arrows represent vacuoles in cells, yellow arrows represent nuclear pyknosis, red arrows represent hemosiderin exudation, and green arrows represent syncytia. White scale bar = 100 µm.

**Figure 5:**
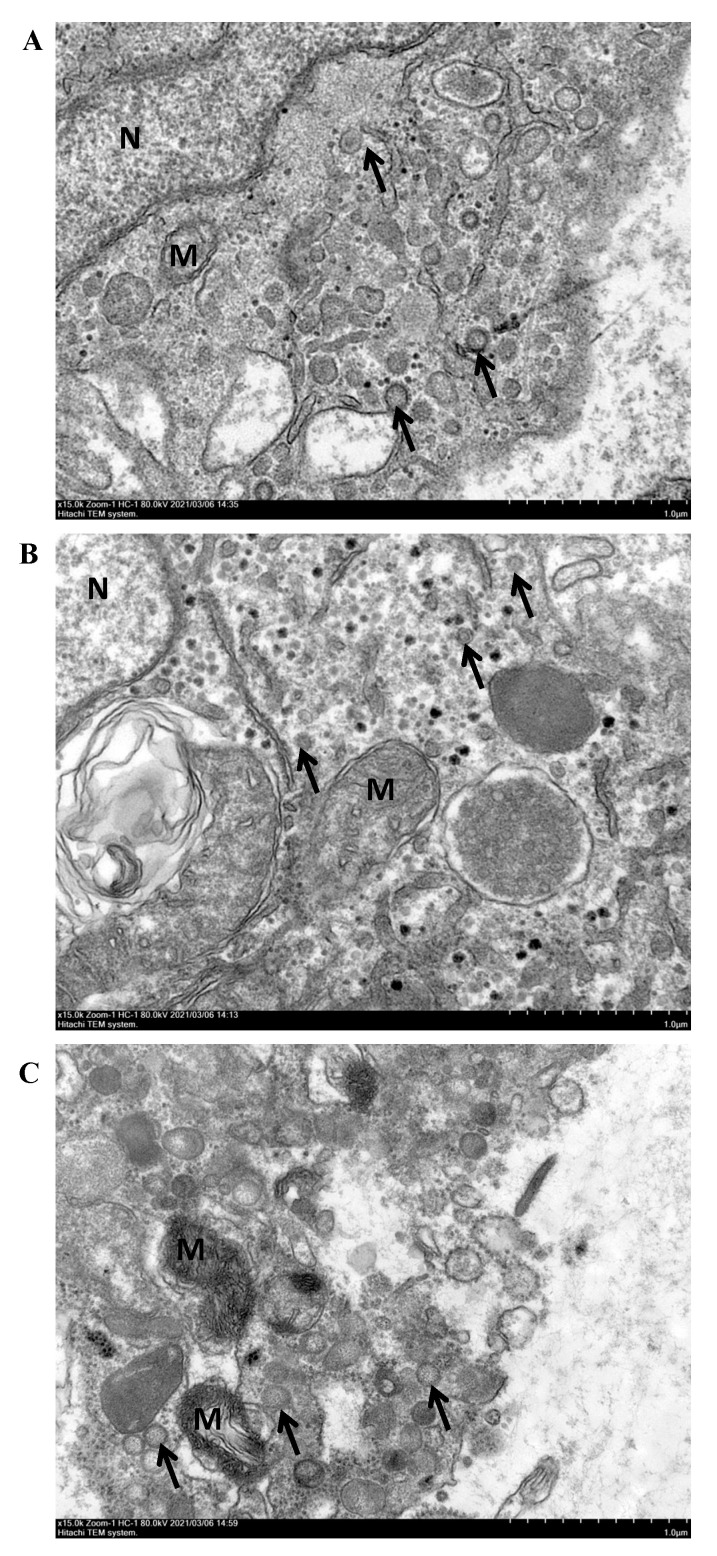
Transmission electron microscope observation of different tissues from the American eels infected with AngHV-1-FC. Virus particles were observed in the gill (**A**), liver (**B**) and spleen (**C**). Black arrows show virus particles within the cytoplasm. N, nucleus. M, mitochondrion. Scale bar = 100 nm.

The histopathological examination showed that an increase in the vacuoles could be observed in gill, liver and spleen cells (Figure 4). Several cells with nuclear pyknosis were observed in the liver of challenged fish. Some syncytia and increased loci with hemosiderin exudation were also found in the spleen of challenged fish. However, all these symptoms were absent in fish in the control group. Moreover, the DNA polymerase gene sequence of AngHV-1 was also detected from the lesioned tissues of artificially infected eels, which was consistent with the results of PCR detection in naturally diseased eels (Supplementary Materials, Figure S1). Then, the viral copy number was determined to be 4200 ± 600 copies/µL DNA sample from the fish in the infection group by quantitative PCR, while the viral genome was not detected in the fish in the control group (Supplementary Materials, Figure S4). TEM observation showed several virus particles within the cytoplasm of cells in the gills, liver and spleen at 14 dpi (Figure 5). These results indicated that AngHV-1-FC was the pathogen causing hemorrhagic disease in the farmed American eels, with a high pathogenicity.

### 3.4. Identification of AngHV-1-FC

**Figure 6:**
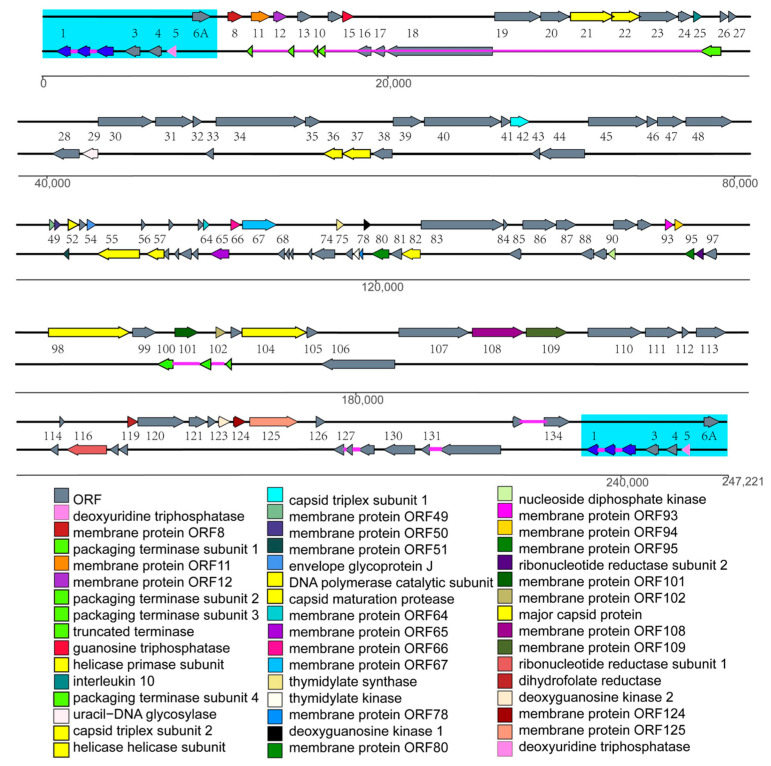
Map of the genome structure of AngHV-1-FC with the protein annotations. The predicated ORFs are indicated by colored arrows and the directions of arrows represent transcription initiation direction of them. ORFs are depicted as color-shaded arrows, with names (lacking the ORF prefix) between the two strands. Introns connecting spliced ORFs are shown as narrow purple bars according to the transcriptome information of the reference genome [15]. The 12 core ORFs conserved in all *Alloherpesviridae* isolates are presented in light green or yellow arrow. The other identical ORFs among three virus isolates FC, FJ and TW are presented in different colors with different functions and the ORFs with unknown functions are presented in grey. The name of ORFs with known functions are shown in the list below the map. The TR is shown in cyan.

**Figure 7:**
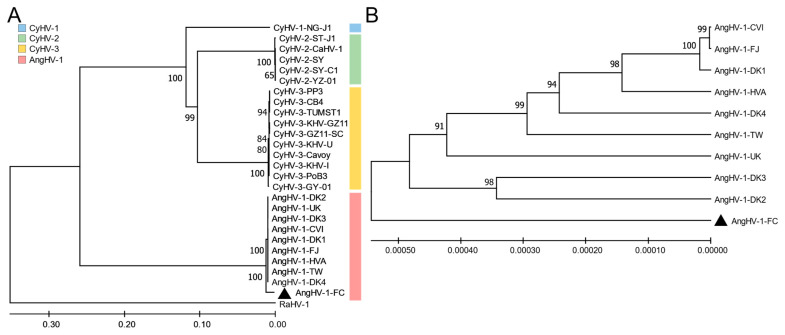
Phylogenetic trees of AngHV-1-FC. (**A**) The phylogenetic tree of concatenated 3 core gene amino acid sequences (DNA polymerase, DNA helicase, and terminase genes from AngHV-1, *Cyprinivirus* and RaHV-1 genomes). The tree was constructed by the neighbor-joining method with 1000 bootstrap replicates. (**B**) The phylogenetic tree based on whole genome sequences of AngHV-1 isolates. The tree was constructed by the UPGMA method. Only values greater than 50% are shown. The AngHV-1-FC is marked by a black triangle.

To determine the taxonomy of AngHV-1-FC, we sequenced its whole genome. After quality control and assembly, the AngHV-1-FC genome (Accession no. OM649903.1) was 247,221 bp in length, including a 10,346 bp terminal direct repeat (TR) (Figure 6). The AngHV-1-FC genome encoded 126 ORFs, including core ORFs conserved in all *Alloherpesviridae* isolates (Supplementary Materials, Figure S5, Table S3). These core ORFs encoded proteins involved in capsid morphogenesis, DNA replication, and DNA packaging [14,15]. The ANI analysis showed that the ANI values between AngHV-1-FC and other AngHV-1 isolates ranged from 99.28% to 99.55%, whereas the ANI values between AngHV-1-FC and 3 *Cyprinivirus* isolates ranged from 62.36% to 66.17% (Table 1). Moreover, the phylogenetic tree based on the concatenated alignment of amino acid sequences of three core genes showed that AngHV-1-FC and other AngHV-1 isolates were clustered into a group, while all the *Cyprinivirus* isolates were clustered into another group (Figure 7A). Similarly, AngHV-1-FC was also assigned to the AngHV-1 group according to the phylogenetic tree analysis based on the whole genomes, confirming that AngHV-1-FC was a member of AngHV-1 (Figure 7B).

However, it is worth noting that AngHV-1-FC was in the outermost branch of the AngHV-1 groups in two phylogenetic trees. The mean value of the evolutionary divergence between the genome sequences of AngHV-1-FC and other AngHV-1 isolates was 1.35 × 10^−3^, which was more than that among other AngHV-1 isolates (0.83 × 10^−3^) (Table 2). A comparative analysis of homologous genes showed that 125 ORFs of AngHV-1-FC shared higher sequence identities (>95%) with the corresponding ORFs of reported AngHV-1 genomes. However, the ORF90L of AngHV-1-FC, a predicted nucleoside diphosphate kinase gene, shared only 83.74% sequence identity with the ORF90 of AngHV-1 (YP_003358229.2) (Supplementary Materials, Table S3). These results indicated that AngHV-1-FC was a new isolate of AngHV-1 from the American eels.

## 4. Discussion

Hemorrhagic disease caused by AngHV-1 is one of the most severe diseases affecting both wild and farmed eels [3,4]. In the last two decades, there have been frequent outbreaks of this viral disease in European eels, Japanese eels, giant mottled eels, and shortfin eels [6,7,9,10]. AngHV-1 was also believed to be the main cause of the decline in the European eel, thus European eels are now classified as a critically endangered species [30]. As the main culture species at present, the American eel has been reported to be a potential host of AngHV-1. Kempter et al. (2014) detected AngHV-1 by PCR in imported frozen American eel products [8]. In addition, Zhuo (2015) also detected AngHV-1 by PCR in farmed American eels with “red liver disease”, which showed symptoms similar to hemorrhagic disease [9]. Viral particles of herpesvirus were also observed in lesions in gills by TEM [31]. However, there was no direct evidence that AngHV-1 causes hemorrhagic disease in American eels.

In this study, we isolated an AngHV-1 isolate (AngHV-1-FC) from farmed American eels with suspected hemorrhagic disease in Longyan, China. The diseased fish displayed congestion in the fins and jaw, severe anemia of the gills and liver and enlargement of the spleen. These symptoms were similar to those of the hemorrhagic disease caused by AngHV-1 in farmed Japanese eels, European eels, and giant mottled eels [2,5,10,11]. The DNA polymerase gene of AngHV-1 wase detected in the lesions of the diseased American eels, which shared 100% nucleotide sequence identity with the homologous genes of multiple AngHV-1 isolates from European eels, Japanese eels and giant mottled eels [10,14,15,16,17,32]. Furthermore, the homogenates of lesion tissue elicited CPE in EES cells, resulting in the rounding of cells and the appearance of syncytia. These morphological changes in the EES cells were the same as those observed in AngHV-1-infected eel ovary cells (EO) and two European eel kidney cells [11,12,19]. The viral particles of AngHV-1-FC were also observed in infected EES cells, showing the typical concentric circular morphology of AngHV-1 [11,12]. Meanwhile, several types of viral particles in various stages of intracellular development also confirmed the replication of AngHV-1-FC in EES cells. These results suggest that the AngHV-1-FC may cause hemorrhagic disease in American eels.

Experimental infection in vivo is an effective means of identifying a pathogen, but few studies have investigated the pathogenicity of AngHV-1 isolates under experimental conditions [11,12,33]. In the present study, the artificial injection of AngHV-1-FC with a dose of 1.26 × 10^5^ TCID_50_ per fish caused typical symptoms of hemorrhagic disease in the American eels, which were the same as those observed in naturally diseased American eels and in AngHV-1-infected European eels and Japanese eels [11,12,31,33]. AngHV-1-FC could be detected in the artificially infected eels by PCR and TEM, further confirming that AngHV-1-FC was the pathogen in the hemorrhagic disease in farmed American eels. In addition, the eels infected with AngHV-1-FC showed 100% cumulative morbidity and 13.3% cumulative mortality during 28 d infection. Tatsuya et al. (1997) reported that a AngHV-1 isolate from Japanese eels caused severe hemorrhagic lesions in the skin at an injection dose of 1.26 × 10^7^ TCID_50_ per fish, but had no lethal effect on eels during 14 days post-injection [33]. The AngHV-1 isolate HVA, isolated from European eels, only induced 15% cumulative morbidity but did not produce mortality using an immersion challenge dose of 7.76 × 10^4^ TCID_50_ [12]. However, another AngHV-1 isolate (NA16108) from European eels can cause up to 63.3% cumulative mortality with an injection dose of 10^6^ PFU (approximately 7.08 × 10^5^ TCID_50_) per fish during 28 dpi [11]. The AngHV-1 isolates exhibited different abilities to grow in vitro based on a comparison of the replication fitness of the six AngHV-1 isolates (DK2, DK3, DK4, CVI, UK, and HVA) in EK-1 cells [17]. These data suggested that the pathogenicity varied among AngHV-1 isolates. The AngHV-1-FC had a high pathogenicity, as it could infect adult American eels and the cumulative morbidity was up to 100% with the typical symptoms, even with a lower injection dose (1.26 × 10^5^ TCID_50_ per fish). Moreover, the clinical symptoms were more prevalent in the stressed eels subjected to AngHV-1-FC infection. However, a previous report showed that AngHV-1 infection was only detected in the diseased glass American eels by PCR, with milder symptoms or a red liver [9].

To date, several AngHV-1 isolates have been isolated from different species of eels [3,14,16]. In comparing the genomes of nine AngHV-1 isolates, Donohoe et al. (2021) found low genetic diversity between the AngHV-1 isolates [17]. In this study, the genome of AngHV-1-FC was sequenced and compared with that of nine reported AngHV-1 isolates. The ANI analysis showed a genome identity value of 99% between AngHV-1-FC and the other AngHV-1 isolates, indicating there was a much lower genetic diversity among them. However, phylogenetic analysis showed AngHV-1-FC was in the outermost branch of the AngHV-1 groups in both phylogenetic trees based on three core genes and whole genomes, suggesting there was evolutionary divergence between AngHV-1-FC and the other nine AngHV-1 isolates. Further analysis showed that the nucleoside diphosphate kinase of AngHV-1-FC shared only 83.74% amino acid sequence identity with the homologous protein of AngHV-1, although the sequence identities of other homologous genes between AngHV-1-FC and other AngHV-1 isolates were more than 95%. Nucleoside diphosphate kinase (NDK) is a highly conserved enzyme that is ubiquitous in cellular organisms and viruses [34,35,36]. NDK catalyzes the production of nucleoside triphosphates (NTPs) from nucleoside diphosphates (NDPs), which is central to the synthesis of DNA and RNA [36]. Therefore, these data indicate that AngHV-1-FC is a new isolate of AngHV-1 from farmed American eels, with a high pathogenicity, thus providing new knowledge in regard to the prevalence and prevention of AngHV-1.

## Figures and Tables

**Table 1:** Average nucleotide identity (ANI) analysis of AngHV-1-FC genome.

Isolates	ANIb (%)
AngHV-1-FJ	99.44
AngHV-1-CVI	99.44
AngHV-1-HVA	99.43
AngHV-1-DK1	99.46
AngHV-1-DK2	99.28
AngHV-1-DK3	99.36
AngHV-1-DK4	99.55
AngHV-1-UK	99.41
AngHV-1-TW	99.28
CyHV-1-NG-J1	62.36
CyHV-2-ST-J1	66.17
CyHV-3-KHV-U	63.39

**Table 2:** Estimates of evolutionary divergence between genome sequences of AngHV-1 isolates.

	FC	CVI	DK1	FJ	HVA	TW	DK4	UK	DK3
CVI	1.37 × 10^−3^								
DK1	1.35 × 10^−3^	3.64 × 10^−5^							
FJ	1.54 × 10^−3^	4.05 × 10^−6^	3.24 × 10^−5^						
HVA	1.18 × 10^−3^	3.20 × 10^−4^	3.24 × 10^−4^	3.40 × 10^−4^					
TW	1.68 × 10^−3^	6.73 × 10^−4^	6.69 × 10^−4^	9.16 × 10^−4^	5.18 × 10^−4^				
DK4	1.09 × 10^−3^	6.17 × 10^−4^	6.14 × 10^−4^	6.21 × 10^−4^	4.34 × 10^−4^	7.96 × 10^−4^			
UK	1.40 × 10^−3^	9.93 × 10^−4^	9.97 × 10^−4^	1.03 × 10^−3^	8.70 × 10^−4^	1.18 × 10^−3^	8.95 × 10^−4^		
DK3	1.24 × 10^−3^	1.07 × 10^−3^	1.06 × 10^−3^	1.14 × 10^−3^	9.24 × 10^−4^	1.29 × 10^−3^	9.08 × 10^−4^	1.11 × 10^−3^	
DK2	1.28 × 10^−3^	1.22 × 10^−3^	1.22 × 10^−3^	1.28 × 10^−3^	1.13 × 10^−3^	1.50 × 10^−3^	1.11 × 10^−3^	1.30 × 10^−3^	7.80 × 10^−4^
Mean value	1.35 × 10^−3 #^		8.31 × 10^−4^ *						

# This value was calculated from the estimates of evolutionary divergence between AngHV-1-FC and other nine AngHV-1 isolates. * This value was calculated from all estimates of evolutionary divergence among the nine AngHV-1 isolates.

## Data Availability

The data presented in this study are available on request from the corresponding author.

## Supplementary Materials

The following supporting information can be downloaded at: https://www.mdpi.com/article/10.3390/v14122722/s1, Figure S1: The detection of AngHV-1 in naturally diseased or artificially infected eels; Figure S2: The DNA polymerase gene segment of the AngHV-1-FC; Figure S3: The symptoms of the American eels infected with AngHV-1-FC; Figure S4: The viral copy number in fish of control group or infection group at 28 dpi; Figure S5: The circular representation of AngHV-1-FC; Table S1: The isolates with the accession no. used for construction of the phylogenetic tree; Table S2: The sequences identity of DNA polymerase gene of AngHV-1-FC with different AngHV-1 isolates; Table S3: The complete genome annotation of AngHV-1-FC.
