# Supplementary material for: Isolation and Identification of a New Isolate of Anguillid Herpesvirus 1 from Farmed American Eels (Anguilla rostrata) in China
Source: Viruses. 2022 Dec 7;14(12):2722. doi: 10.3390/v14122722 (PMC9784739; doi:10.3390/v14122722)
Supplement: Supplementary file 1 [file viruses-14-02722-s001.zip › 1205-Supplementary Figures.docx]

**
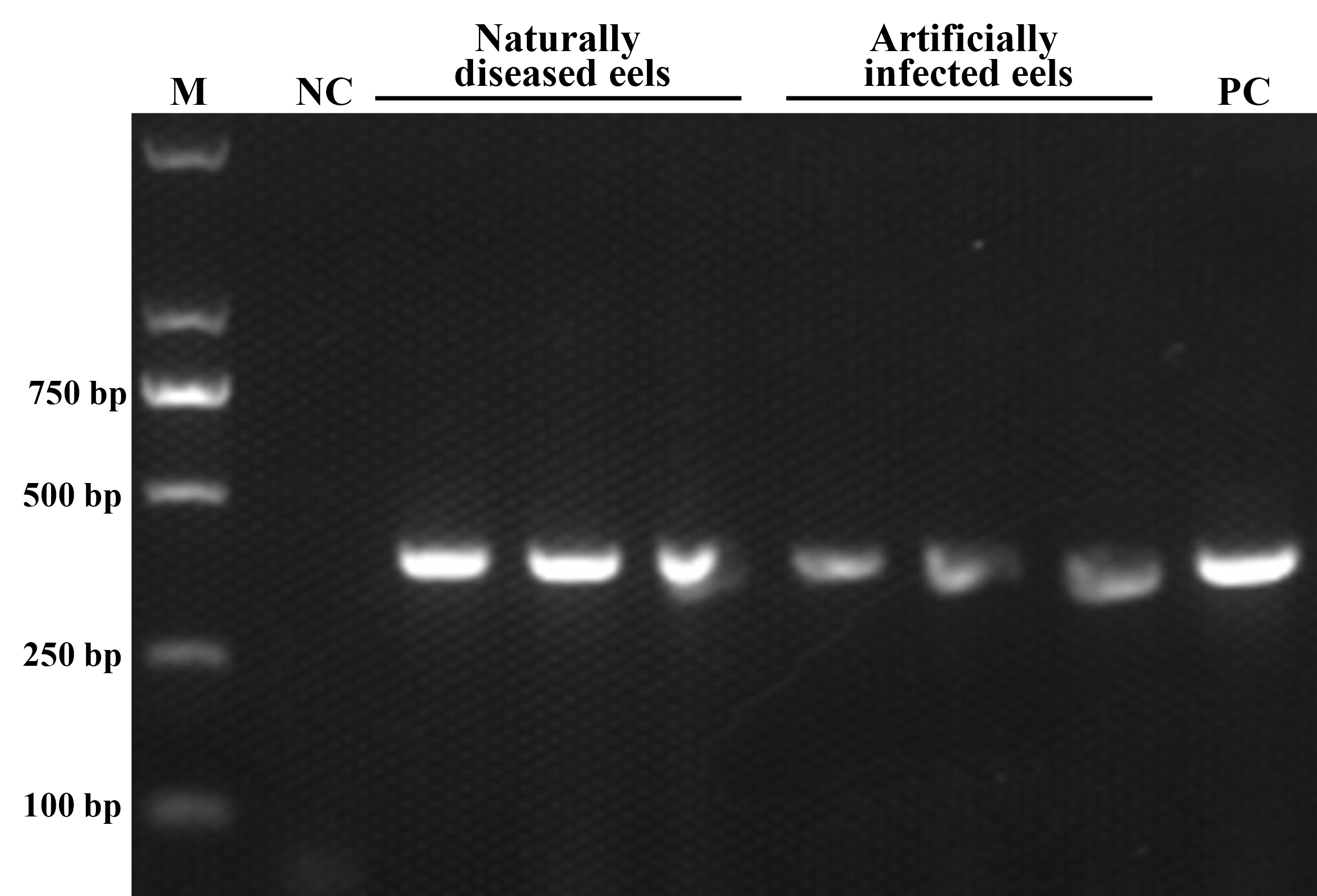
**

**Supplementary Materials Figure S1.** The detection of AngHV-1 in naturally diseased or artificially infected eels. The amplicons of AngHV-1 DNA polymerase gene (394 bp) from naturally affected eels, artificially infected eels, and healthy eels by PCR. M, molecular marker. NC, negative control. PC, positive control.

**
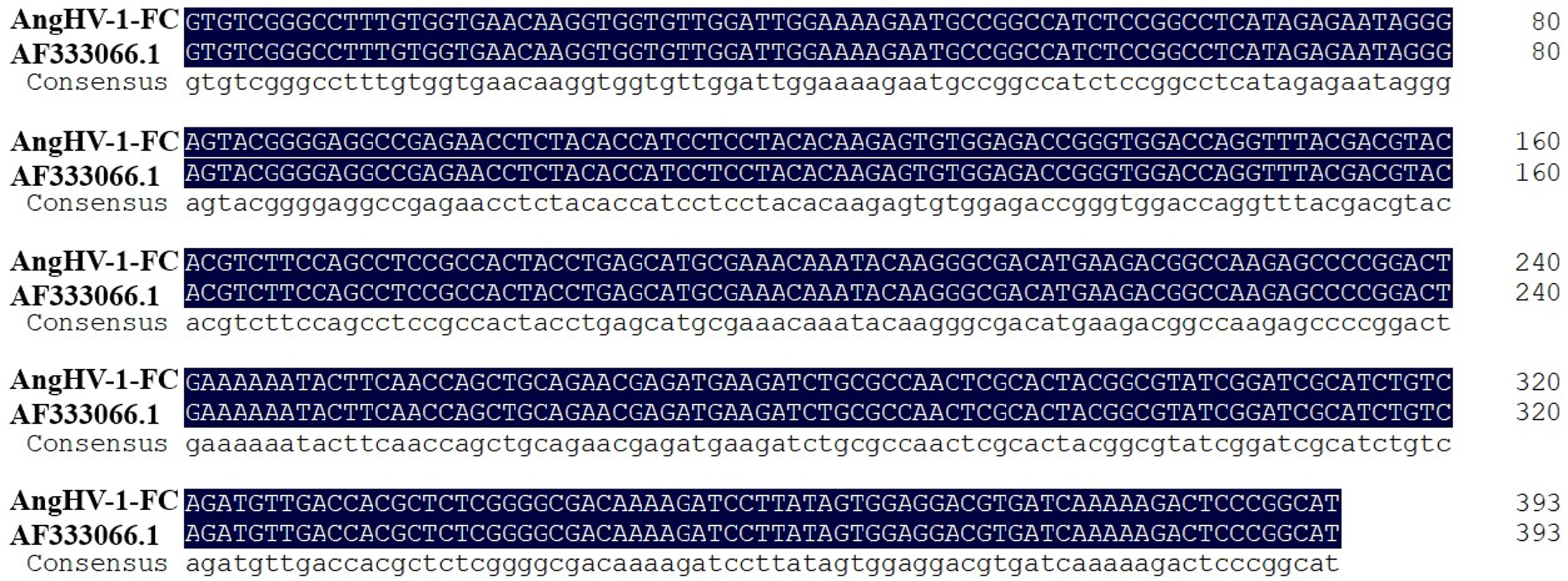
**

**Supplementary Materials Figure S2.** The DNA polymerase gene segment of the AngHV-1-FC showed 100% nucleotide sequence identity with that of AngHV-1 (GenBank accession no. AF333066.1).

**
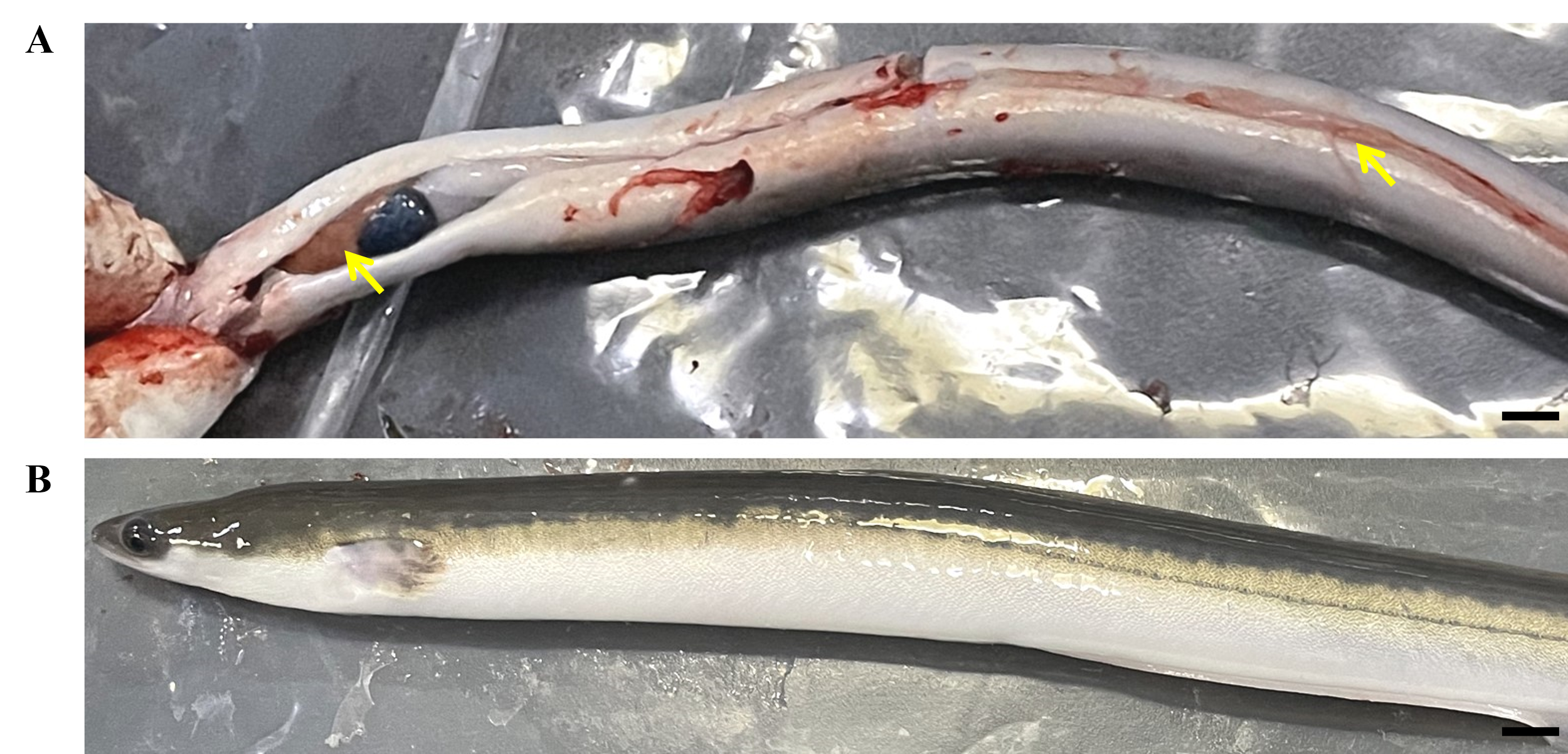
**

**Supplementary Materials Figure S3.** The symptoms of the American eels infected with AngHV-1-FC. (**A**) The eel in infection group at 10 dpi. The yellow arrows showed the ischemic liver and congestions of anal fin. Scale bar=1 cm. (**B**) The eel in control group at 10 dpi. There was no clinical symptom of infection. Scale bar=1 cm.

**
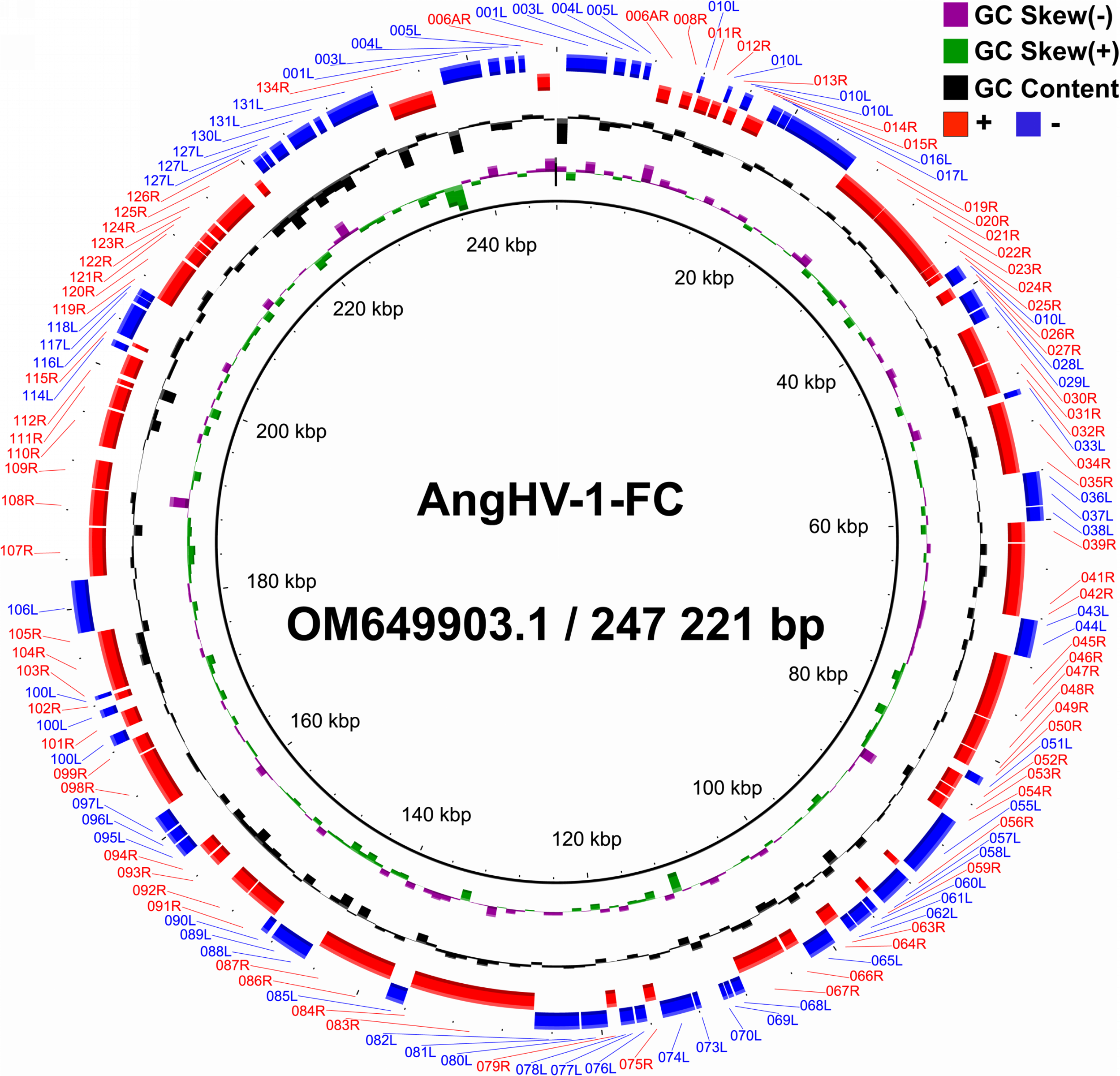
**

**Supplementary Materials Figure S4.** The circular representation of AngHV-1-FC. From outside inwards, the rings show ORFs on the positive strand (red), or on the negative strand (blue), GC content for the positive and negative strands (black), and GC skew for the positive (green) and negative (purple) strands.
